# Context-dependent effects of the CB1 receptor antagonist rimonabant on morphine-induced behavioral sensitization in female mice

**DOI:** 10.3389/fphar.2023.1100527

**Published:** 2023-02-06

**Authors:** Eduardo A. V. Marinho, Alexandre Justo Oliveira-Lima, Henrique S. Reis, Renan Santos-Baldaia, Raphael Wuo-Silva, Andre W. Hollais, Thais S. Yokoyama, Roberto Frussa-Filho, Lais F. Berro

**Affiliations:** ^1^ Department of Health Sciences, Universidade Estadual de Santa Cruz, Ilhéus, BA, Brazil; ^2^ Department of Pharmacology, Universidade Federal de São Paulo, São Paulo, SP, Brazil; ^3^ Department of Psychiatry and Human Behavior, University of Mississippi Medical Center, Jackson, MS, United States

**Keywords:** morphine, rimonabant, CB1 receptor, environment, context, mice

## Abstract

**Introduction:** The endocannabinoid system has been implicated in the neurobiology of opioid use disorder. While the CB1 receptor antagonist rimonabant has been shown to block some of the behavioral effects of opioids, studies suggest that the treatment environment (i.e., receiving treatment in the drug-associated environment, and/or novelty) can influence its effects. In the present study, we investigated the role of the treatment environment in the effects of rimonabant on the expression of morphine-induced behavioral sensitization.

**Methods:** Adult female Swiss mice were submitted to a behavioral sensitization protocol, during which they received morphine (20 mg/kg, i.p.) in the open-field apparatus, and were subsequently treated with vehicle or rimonabant (1 or 10 mg/kg, i.p.) either in the open-field, in the home-cage or in an activity box (novel environment). The expression of conditioned locomotion (increased locomotor activity in the open-field apparatus in the absence of morphine) and of morphine-induced behavioral sensitization (increased locomotor activity in animals sensitized to morphine) was evaluated during asubsequent saline and morphine challenge, respectively.

**Results:** Animals treated with morphine expressed behavioral sensitization, showing a significant increase in locomotor activity over time. Animals sensitized to morphine and treated with vehicle in the home-cage expressed conditioned locomotion, an effect that was blocked by home-cage treatment with rimonabant. During a saline challenge, only animals sensitized to morphine and treated with saline in the home-cage expressed morphine-induced conditioned locomotion. All morphine-treated animals that received saline during the treatment phase (control groups) expressed behavioral sensitization during the morphine challenge. Treatment with rimonabant in the open-field and in the activity box, but not in the home-cage, blocked the expression of morphine-induced behavioral sensitization.

**Discussion:** Our findings suggest that CB1 receptor antagonism can modulate conditioned responses to morphine even when administered in the home-cage. However, exposure to the drug-associated environment or to a novel environment is necessary for the expression of rimonabant’s effects on morphine-induced behavioral sensitization during a morphine challenge.

## 1 Introduction

Opioid use disorder (OUD) remains a major public health problem worldwide, and an epidemic in the United States. According to the most recent World Drug Report, approximately 61 million people reported opioid use in 2020 (UNODC, 2022). Data from the Centers for Disease Control and Prevention show that the predicted number of overdose deaths involving opioids has increased by 125% from January 2015 to January 2022, with a reported number of opioid-related overdose deaths of 104,034 in the 12 months between February 2021–2022 (Ahmad et al., 2022). According to the latest Global Drug Survey (Winstock et al., 2021), the majority of those who reported using heroin priotirized drug-related pleasure. However, the use of prescription opioids remains high, and recent data indicate that prescription opioid use is significantly higher among women than men (Hales et al., 2020). Several studies have investigated sex differences in opioid use (for review, see Craft, 2008; Serdarevic et al., 2017; Nicolas et al., 2022). Of note, studies suggest that females may be more vulnerable to the reinforcing effects of opioids (Goetz et al., 2021), and that female rats acquire opioid self-administration more rapdly than males (Lynch and Carroll, 1999), emphasizing the importance of research investigating the effects of opioids in female subjects.

Acute treatment with opioids stimulate µ opioid receptors in the ventral tegmental area (VTA), leading to an increased activation of dopaminergic neurons and dopamine levels in the nucleus accumbens (NAcc), and this mechanism mediates opioid reward and reinforcement (Kim et al., 2016; Listos et al., 2019). The activation of the mesocorticolimbic dopaminergic system is related to the rewarding effects exerted by drugs of abuse and observed in the early stages of addiction (Volkow et al., 2016). Of note, the endocannabinoid system has been implicated in the neurobiology of OUD, and CB1 receptors located in the VTA and in the NAcc mediate the actions of endocannabinoids (Mechoulam et al., 1996). In fact, studies suggest that an interaction exists between endocannabinois and opioid systems in reward-related behaviors, particularly *via* CB1 receptors. For instance, intra-NAcc (Azizi et al., 2009) and intra-VTA (Rashidy-Pour et al., 2013) administration of a CB1 receptor antagonist blocked the acquisition and expression of morphine-induced conditioned place preference.

Studies suggest that the effects of CB1 receptors on drug abuse seem to be modulated by drug-environment conditioning (Gerdeman et al., 2008). In fact, we have shown that the effects of the CB1 receptor antagonist rimonabant on ethanol-induced conditioned place preference were context-dependent (i.e., the treatment blocked the expression of conditioned place preference to ethanol when given in the drug-associated enrivonment, but not when given in a saline-paired environment) (Silva et al., 2017). The context-dependent effects of rimonabant have been attributed to the activity of the cannabinoid signaling as an occasion setter (Gerdeman et al., 2008; Silva et al., 2017). Studies have shown that midbrain dopamine neurons act as a neurobiological substrate for encoding occasion setting properties (Aquili et al., 2020). Of note, our studies also showed that rimonabant blocked the development of rapid-onset behavioral sensitization to morphine (Marinho et al., 2017), suggesting that endocannabinoids also mediate opioid-induced sensitization. However, it remains unknown whether CB1 antagonism plays a major role in the expression of morphine-induced sensitization, and whether the treatment environment mediates these effects.

In the present study, we investigated the potential role of CB1 receptors in modulating morphine-induced behavioral sensitization. Importantly, the CB1 receptor antagonist rimobanat was administered after a sensitization protocol in three distinct environmental contexts: the drug-associated environment, the home-cage and a novel environment. These studies allowed us to investigate the role of the treatment environment in the effects of rimonabant on the expression of morphine-induced behavioral sensitization.

## 2 Materials and methods

### 2.1 Subjects

Female three-month-old Swiss EPM-M1 mice (30–35 g) from our own colony were used. The animals were group housed (7 animals per cage) in standard polypropylene cages (32 cm × 42 cm x 18 cm) under conditions of controlled temperature (22°C–23°C) and lighting (12/12 h light/dark, lights on at 06:45 h). Food and water were available *ad libitum* throughout the experiments, except during the 10-min behavioral sessions, when animals were exposed to the open-field apparatus or the activity box (novel environment). The experiments were performed in accordance with the National Institute of Health Guide for Care and Use of Laboratory Animals (NIH Publications No 8023, revised in 2011) and the Brazilian Law for Procedures for Animal Scientific Use (#11794/2008). The Institutional Animal Care and Use Committee of UNIFESP approved the experimental procedures under protocol #470/07. The three different experiments were conducted with separate cohorts of naïve animals.

### 2.2 Drugs

Morphine (20 mg/kg, SIGMA^®^) was freshly diluted in 0.9% NaCl (Saline) solution. Saline was used as the control solution. Rimonabant (1 or 10 mg/kg, SR141716; Sanofi-Aventis^®^, Paris, France) was dissolved in 1% Tween 80 and then diluted in distilled water. Solution of 1% Tween 80 in distilled water was used as the rimonabant control (Vehicle). The solutions were administered intraperitoneally (i.p.) at a volume of 10 mL/kg body weight.

### 2.3 Open-field evaluation

Locomotor activity was measured in the open-field. The open-field apparatus consisted of a circular wooden floor (40 cm in diameter and 50 cm high) with white acrylic walls and an open top. The floor was subdivided into 19 approximately equal regions demarcated by concentric circles of different radii (4, 12, and 20 cm), intersected by segments of radial lines. During a behavioral session, the open-field apparatus was placed on the floor of an experimental room, which was temperature controlled (22°C–23°C). In order to acclimate to the experimental room, the animals’ home-cages were placed in the experimental room at least 1 h before behavioral sessions. Using hand-operated counters and stopwatches, the locomotion frequency (i.e., total number of times the animal crossed a line dividing the floor segments) was measured by an observer (who was blinded to the treatment allocation) during a 10-min session. Evaluation of mouse behavior in the open-field was conducted for 10 min (Oliveira-Lima et al., 2015). During the 10-min open-field sessions, animals did not receive food or water. After each animal was removed from the apparatus, the open-field was cleaned with a 5% alcohol/water solution to minimize any olfactory influences on the next behavioral session.

### 2.4 Experimental procedure

**FIGURE 1:**
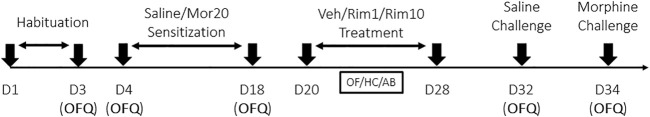
Design of experiments 1–3. OFQ, Open-Field Quantification of locomotor activity; Mor20, Morphine (20 mg/kg, i.p.); Veh, Vehicle (i.p.); Rim1, Rimonabant (1 mg/kg, i.p.); Rim10, Rimonabant (10 mg/kg, i.p.); OF, Open-field (drug-associated environment); HC, home-cage; AB, activity box (novel environment). Sensitization: every other day treatment with morphine or saline. Treatment: daily treatment with vehicle or rimonabant in the open-field (Experiment 1), in the home-cage (Experiment 2) or in the activity box (Experiment 3).

The experimental design for Experiments 1–3 is summarized in Figure 1. All treatments and behavioral sessions were conducted during the light phase.

#### 2.4.1 Experiment 1: Effects of treatment with rimonabant in the drug-associated environment (open-field) on morphine-induced behavioral sensitization

##### 2.4.1.1 Morphine sensitization phase

The experimental design was performed according to a protocol previously developed by our group (Oliveira-Lima et al., 2015). Seventy-two female mice were exposed to the open-field apparatus (10-min sessions) for three consecutive days for habituation, and their total locomotor frequency was measured on the third day. During the habituation sessions, all animals received saline (Sal) injections. Thirty min after treatments, animals were placed individually in the apparatus for 10 min. After habituation, mice were allocated to six experimental groups (*n* = 14 per group) based on similar total locomotor activity frequencies (groups Sal-Veh; Sal-Rim1, Sal-Rim10, Mor-Veh, Mor-Rim1, and Mor-Rim10). Starting on Day 4, animals received either an intraperitoneal injection (i.p.) of saline (Sal-) or 20 mg/kg morphine (Mor-) every other day for 15 days (total of eight treatments, Days 4–18). Thirty minutes after each treatment, animals were exposed to a 10-min open-field session. Locomotor activity was measured on the first and last (15th) days of the sensitization protocol.

##### 2.4.1.2 Rimonabant treatment phase

48 h after the last morphine injection, the rimonabant treatment phase begun. Mice received an i.p. injection of Vehicle (Sal- or Mor-Veh), 1 mg/kg rimonabant (Sal- or Mor-Rim1) or 10 mg/kg (Sal- or Mor-Rim10) for eight consecutive days (Days 20–28). Animals were exposed to a 10-min open-field session 30 min after the injection. Locomotor activity was not measured during this phase.

##### 2.4.1.3 Saline challenge

Four days after the last treatment (Day 32), all animals received an acute i.p. Saline injection and, 5 min after injection, were exposed to a 10-min open-field session during which locomotor activity was measured.

##### 2.4.1.4 Morphine challenge

Two days after the saline challenge (Day 34), all animals received an acute i.p. injection of morphine (20 mg/kg) and, 30 min after injection, were exposed to a 10-min open-field session during which locomotor activity was measured.

The pretreatment time for morphine during the sensitization phase was determined based on pharmacokinetic studies in mice showing that plasma and brain levels of morphine reached a peak 15 and 45 min after an i.p. injection of 17.8 mg/kg morphine, respectively (Koek et al., 2012). Pretreatment time was also determined based on previous studies from our laboratory showing that a 30 min pretreatment is effective in inducing behavioral sensitization to morphine in mice (Hollais et al., 2014; Marinho et al., 2015; Trombin et al., 2018). The same pretreatment time used during the sensitization phase (30 min) was used for the morphine challenge. For the saline challenge, an injection was used simply to control for an additional drug-associated manipulation. Because saline is an inert substance, the saline challenge was performed with a 5 min pretreatment time. As shown in our results, the control group that never received morphine or rimonabant (saline-vehicle group) showed similar locomotor activity during the saline challenge compared to itself during the habituation and sensitization phases, during which a 30 min pretreatment time was used. Therefore, a shorter pretreatment time during the saline challenge did not affect the animals’ locomotor activity.

The doses of morphine and rimonabant were based on previous studies from our laboratory using similar procedures in mice (Hollais et al., 2014; Marinho et al., 2015; Marinho et al., 017; Trombin et al., 2018). Locomotor sensitization to drugs, including morphine, is a dose-dependent phenomenon (Powell and Holtzman, 2001). In the present study, our aim was to investigate the effects of a post-sensitization treatment on the expression of morphine-induced behavioral sensitization. Therefore, we chose to use a dose of morphine that had been previously shown to induce locomotor sensitization in mice (Zhao et al., 2004). Using a lower or higher dose that does not induce locomotor sensitization would have prohibited us from evaluating the expression of this phenomenon. Of note, our protocol also allowed for the evaluation of the expression of morphine-induced behavioral sensitization after a treatment phase with rimonabant, but in the absence of rimonabant. The last rimonabant treatment was conducted 6 days before the morphine challenge, which guaranteed that rimonabant has been metabolized and, therefore, minimized potentially confounding behavioral effects that could influence our results.

#### 2.4.2 Experiment 2: Effects of treatment with rimonabant in the home-cage on morphine-induced behavioral sensitization

Experiment 2 followed the same protocol for Experiment 1, except during the Rimonabant Treatment Phase, vehicle or rimonabant were administered in the home-cage (animals received an injection and were immediately placed back in their home-cages) instead of in the open-field. Home-cages and the colony room are described in item 2.1, and home-cages remained in the colony room during/after injections. Therefore, animals had access to food and water *ad libitum* and remained group housed before and after drug administrations. Animals were housed with mice in the same treatment group. Therefore, all animals within a cage received the same experimental treatment. Animals did not have access to enrichment objects in the home-cage during the treatment phase.

During the treatment phase of Experiment 2, animals were only handled once/day, for injections. This is an important difference compared to Experiments 1 and 3, during which animals were handled twice/day, for injections and for placement in the behavioral apparatus. However, before the treatment phase, animals had been handled for at least 11 days during the habituation and sensitization phases, which were the same for animals in all experiments. Therefore, any influences of handling during the treatment phase on the results of Experiment 2 vs. Experiments 1 and 3 should be minor.

#### 2.4.3 Experiment 3: Effects of treatment with rimonabant in a novel environment (activity box) on morphine-induced behavioral sensitization

Experiment 3 followed the same protocol for Experiment 1, except during the Rimonabant Treatment Phase, vehicle or rimonabant were administered in an activity box (instead of in the open-field) 30 min after the injection for 10 min. The activity box consisted of a rectangular box (50 cm × 48 cm x 50 cm) with an open top, white walls and an acrylic floor. Activity boxes were placed in a different experimental room compared to Experiment 1. However, the experimental room was in the same building and very similar, and was also temperature controlled (22°C–23°C). In order to acclimate to the experimental room, the animals’ home-cages were placed in the experimental room at least 1 h before behavioral sessions. During the 10-min activity box sessions, animals did not receive food or water. After each animal was removed from the apparatus, the open-field was cleaned with a 5% alcohol/water solution to minimize any olfactory influences on the next behavioral session.

### 2.5 Statistical analysis

Before conducting the parametric tests, locomotor activity data were checked for normality (Shapiro—Wilk test) and homogeneity (Levene’s test), which validated the use of the parametric test. Data were analyzed by one or two-way ANOVA, with or without repeated measures (RM), and multiple comparisons were performed using the Bonferroni’s *post hoc* test when necessary. A *p*-value < 0.05 was considered as a statistically significant difference. All analyses, as well as all graphical representations, were performed using the GraphPad Prism software (version 9).

## 3 Results

### 3.1 Experiment 1: Effects of treatment with rimonabant in the drug-associated environment (open-field) on morphine-induced behavioral sensitization

**FIGURE 2:**
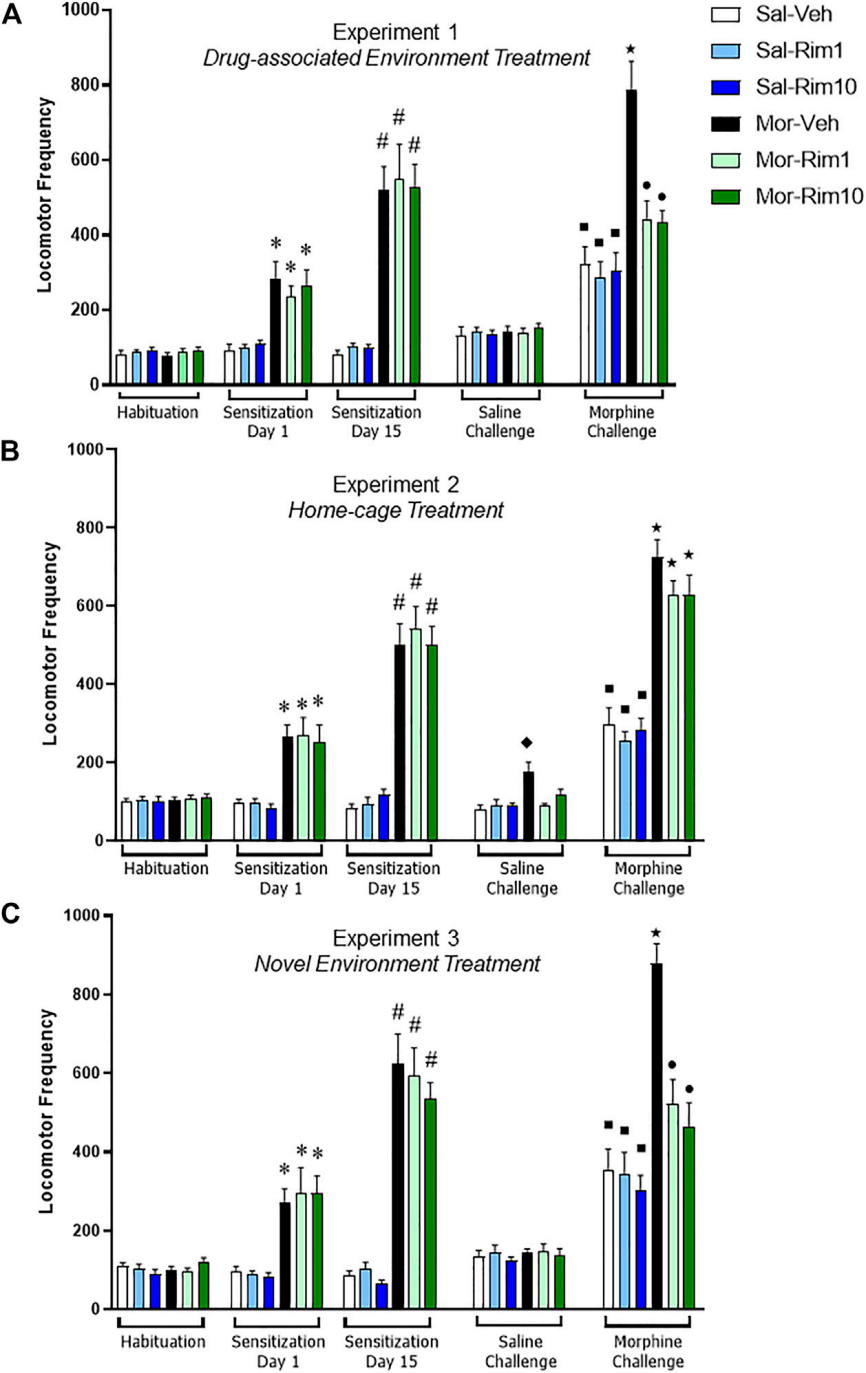
Open-field locomotor activity quantification during the three behavioral experiments. Locomotor activity demonstrating acute hyperlocomotion induced by morphine (Mor, 20 mg/kg) (Sensitization Day 1) and morphine-induced behavioral sensitization (Sensitization Day 15) after a 15-day intermittent treatment (8 morphine or saline—Sal—injections). Effects of treatment with vehicle (Veh) or rimonabant (Rim1 and Rim10, 1 and 10 mg/kg, respectively) for 8 days in the drug-associated environment [**(A)** open-field, Experiment 1], in the home-cage [**(B)** Experiment 2] or in a novel environment [**(C)** activity box, Experiment 3] on subsequent Saline and Morphine challenges. Data are reported as mean ± SEM. *p < 0.05 compared with Saline-treated groups on Sensitization Day 1; #*p* < 0.05 compared with the same group on Sensitization Day 1; ʋp<0.05 compared with the Sal-Veh group on the Saline Challenge; ■*p* < 0.05 compared with the same group during the Saline Challenge; ★*p* < 0.05 compared with the respective control group during the Morphine Challenge; ●*p* < 0.05 compared with Mor-Veh group during the Morphine Challenge.

Data from Experiment 1 are illustrated in Figure 2A. Analysis of the third habituation session revealed that there was no significant difference between groups (one-way ANOVA, six groups: [F (5, 66) = 0.36, *p* = 0.86]). For the morphine sensitization phase, two-way RM ANOVA (factors: treatment—saline vs. morphine; time—Day 1 vs. Day 15 of sensitization) revealed a significant interaction between time and treatment [F (10, 132) = 13.76; *p* < 0.0001]. Bonferroni’s *post hoc* comparisons indicated that morphine induced hyperlocomotion (Mor > Sal, Day 1, *p* < 0.05), an effect that was sensitized after repeated morphine administration (Day 15 > Day 1 for all morphine groups, *p* < 0.05).

During the saline challenge, a two-way ANOVA (factors: treatment 1—saline vs. morphine; treatment 2—vehicle vs. rimonabant) showed no significant effects (treatment 1 [F (1, 66) = 0.49; *p* = 0.48]; treatment 2 [F (2, 66) = 0.06; *p* = 0.93]; interaction [F (2, 66) = 0.20; *p* = 0.81]).

During the morphine challenge, the two-way ANOVA with the same factors as the saline challenge showed a significant interaction between treatments 1 and 2 [F (2, 66) = 6.75; *p* < 0.001]. Bonferroni’s *post hoc* test revealed that animals sensitized to morphine and treated with vehicle showed increased locomotor activity in response to an acute morphine injection compared to the Sal-Veh group (*p* < 0.05). However, animals sensitized to morphine and treated with rimonabant did not differ from their respective groups (Mor-Rim1 vs. Sal-Rim1 and Mor-Rim10 vs. Sal-Rim10, *p* > 0.05). In fact, animals sensitized to morphine and treated with rimonabant showed decreased locomotor activity compared to the group sensitized to morphine and treated with vehicle (*p* < 0.05). An additional two-way RM ANOVA (factors: treatment 1—saline vs. morphine; treatment 2—vehicle vs. rimonabant, challenge: saline vs. morphine) showed a significant interaction between the three factors [F (2, 66) = 7.32; *p* < 0.01]. Bonferroni’s *post hoc* comparisons showed that animals treated with saline during the sensitization phase showed increased locomotor activity after an acute morphine challenge compared to themselves during the saline challenge, irrespective of previous rimonabant treatment (Sal-Veh, Sal-Rim1 and Sal-Rim10, *p* < 0.05).

### 3.2 Experiment 2: Effects of treatment with rimonabant in the home-cage on morphine-induced behavioral sensitization

Data from Experiment 2 are illustrated in Figure 2B. Analysis of the third habituation session revealed that there was no significant difference between groups (one-way ANOVA, six groups: [F (5, 66) = 0.14; *p* = 0.98]). For the morphine sensitization phase, one-way RM ANOVA (factors: treatment—saline vs. morphine; time—Day 1 vs. Day 15 of sensitization) revealed a significant interaction between time and treatment [F (10, 132) = 16.24; *p* < 0.0001]. Bonferroni’s *post hoc* comparisons indicated that morphine induced hyperlocomotion (Mor > Sal, Day 1, *p* < 0.05), an effect that was sensitized after repeated morphine administration (Day 15 > Day 1 for all morphine groups, *p* < 0.05).

During the saline challenge, a two-way ANOVA (factors: treatment 1—saline vs. morphine; treatment 2—vehicle vs. rimonabant) showed a significant interaction between treatmenrs 1 and 2 [F (2, 66) = 6.35; *p* < 0.01]. Bonferroni’s *post hoc* comparisons indicated that animals sensitized with morphine and treated with vehicle showed increased locomotor activity compared to their respective control group (Sal-Veh, *p* < 0.05). However, animals sensitized with morphine and treated with rimonabant did not differ from their respective control groups (*p* > 0.05).

During the morphine challenge, the two-way ANOVA with the same factors as the saline challenge only showed a significant effect of treatments 1 [F (1, 66) = 141.5; *p* < 0.0001]. Bonferroni’s *post hoc* test revealed that animals sensitized to morphine showed increased locomotor activity in response to an acute morphine injection compared to their respective control groups, irrespective of previous vehicle vs. rimonabant treatment (*p* < 0.05). An additional two-way RM ANOVA (factors: treatment 1—saline vs. morphine; treatment 2—vehicle vs. rimonabant, challenge: saline vs. morphine) showed a significant individual effect of the three factors (treatment 1: [F (1, 66) = 167.0; *p* < 0.0001]; treatment 2: [F (2, 66) = 4.02; *p* < 0.05]; challenge: [F (1, 66) = 412.6; *p* < 0.0001]), and a significant interaction between challenge and treatment 1 [F (1, 66) = 91.85; *p* < 0.0001]. Bonferroni’s *post hoc* comparisons showed that animals treated with saline during the sensitization phase showed increased locomotor activity after an acute morphine challenge compared to themselves during the saline challenge, irrespective of previous rimonabant treatment (Sal-Veh, Sal-Rim1 and Sal-Rim10, *p* < 0.05).

### 3.3 Experiment 3: Effects of treatment with rimonabant in a novel environment (activity box) on morphine-induced behavioral sensitization

Data from Experiment 3 are illustrated in Figure 2C. Analysis of the third habituation session revealed that there was no significant difference between groups (one-way ANOVA, six groups: [F (5, 66) = 1.31; *p* = 0.26]). For the morphine sensitization phase, one-way RM ANOVA (factors: treatment—saline vs. morphine; time—Day 1 vs. Day 15 of sensitization) revealed a significant interaction between time and treatment [F (10, 132) = 17.77; *p* < 00,001]. Bonferroni’s *post hoc* comparisons indicated that morphine induced hyperlocomotion (Mor > Sal, Day 1, *p* < 0.05), an effect that was sensitized after repeated morphine administration (Day 15 > Day 1 for all morphine groups, *p* < 0.05).

During the saline challenge, a two-way ANOVA (factors: treatment 1—saline vs. morphine; treatment 2—vehicle vs. rimonabant) showed no significant effects (treatment 1 [F (1, 66) = 0.44; *p* = 0.50]; treatment 2 [F (2, 66) = 0.51; *p* = 0.59]; interaction [F (2, 66) = 0.02; *p* = 0.97]).

During the morphine challenge, the two-way ANOVA with the same factors as the saline challenge showed a significant interaction between treatments 1 and 2 [F (2, 66) = 7.06; *p* < 0.01]. Bonferroni’s *post hoc* test revealed that animals sensitized to morphine and treated with vehicle showed increased locomotor activity in response to an acute morphine injection compared to the Sal-Veh group (*p* < 0.05). However, animals sensitized to morphine and treated with rimonabant did not differ from their respective groups (Mor-Rim1 vs. Sal-Rim1 and Mor-Rim10 vs. Sal-Rim10, *p* > 0.05). In fact, animals sensitized to morphine and treated with rimonabant showed decreased locomotor activity compared to the group sensitized to morphine and treated with vehicle (*p* < 0.05). An additional two-way RM ANOVA (factors: treatment 1—saline vs. morphine; treatment 2—vehicle vs. rimonabant, challenge: saline vs. morphine) showed a significant interaction between the three factors [F (2, 66) = 6.21; *p* < 0.01]. Bonferroni’s *post hoc* comparisons showed that animals treated with saline during the sensitization phase showed increased locomotor activity after an acute morphine challenge compared to themselves during the saline challenge, irrespective of previous rimonabant treatment (Sal-Veh, Sal-Rim1 and Sal-Rim10, *p* < 0.05).

**FIGURE 3:**
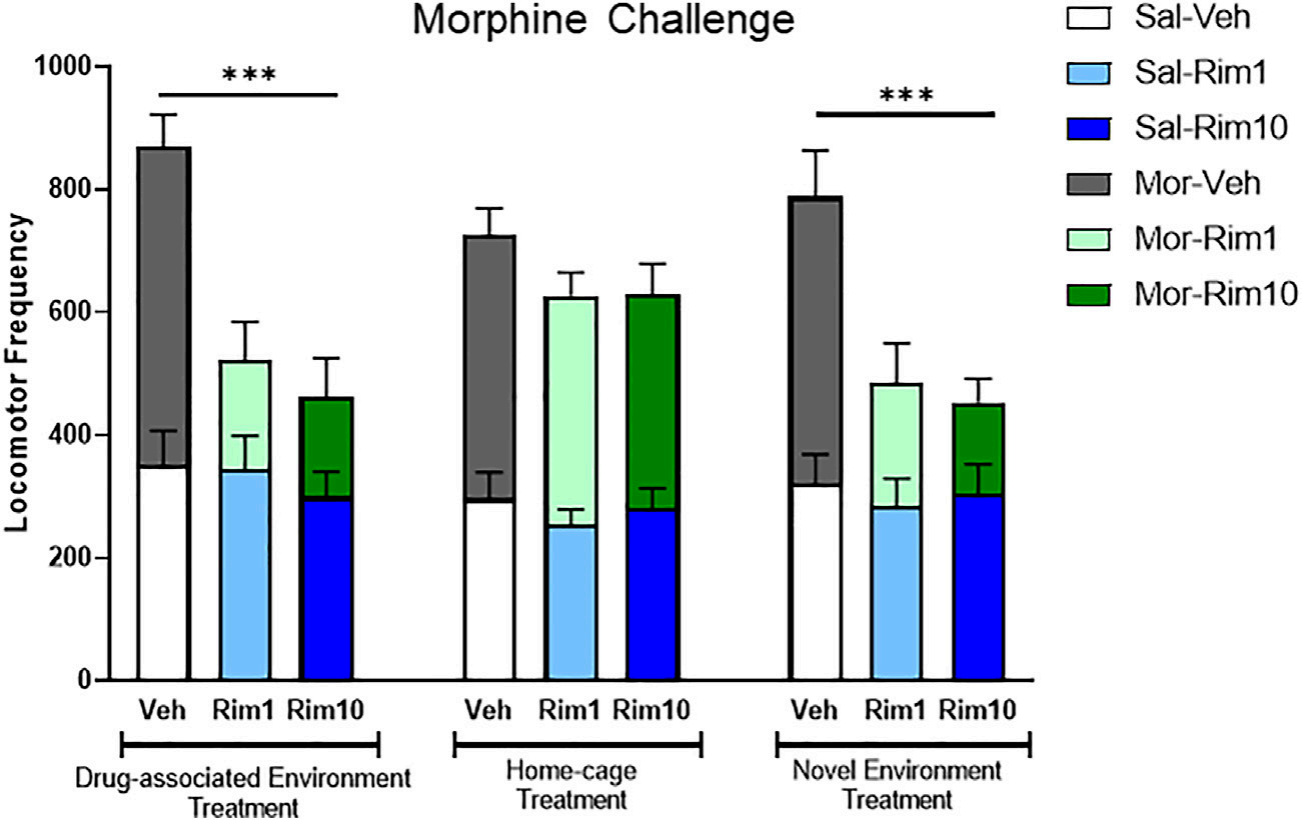
Comparison of the Morphine Challenge data and analyses for all three experiments. Treatment with rimonabant (Rim 1 and Rim 10, 1 and 10 mg/kg, respectively) blocked the expression of morphine-induced behavioral sensitization when animals received treatment in the drug-associated environment (open-field, Experiment 1) or in the novel environment (activity box, Experiment 3), but not when treatment was given in the home-cage (Experiment 2). Veh: vehicle. Data are reported as mean ± SEM and data from saline vs. morphine-sensitized animals are superimposed. ***p < 0.05 compared with the Mor-Veh group during within the same Experiment.

Figure 3 illustrates a comparison of the Morphine challenge data and analyses for all three experiments, indicating that treatment with rimonabant blocked the expression of morphine-induced behavioral sensitization when animals received treatment in the drug-associated environment (open-field) or in the novel environment (activity box), but not when treatment was given in the home-cage.

## 4 Discussion

The present study sought to investigate the role of the treatment environment in the effects of rimonabant on morphine-induced behavioral sensitization. We observed that previous treatment with rimonabant did not block the expression of acute morphine-induced hyperlocomotion (Morphine Challenge, saline-treated animals). However, rimonabant treatment blocked the expression of morphine-induced behavioral sensitization (Morphine Challenge, morphine-treated animals), but only when the animals were exposed to the drug-associated environment or to a novel environment. Finally, animals sensitized with morphine and treated with vehicle in the home-cage showed increased locomotor frequency during the saline challenge compared to the control group (saline-vehicle), indicating the expression of morphine-induced conditioned locomotion (i.e., increased locomotor activity in the drug-associated environment in the absence of morphine). Of note, animals sensitized with morphine and exposed to the open-field apparatus or to the activity box during the treatment phase did not express conditioned locomotion to morphine, suggesting that exposure to the drug-associated environment or to a novel environment led to extinction of morphine-induced conditioned locomotion. Interestingly, while home-cage rimonabant treatment had no effects on the expression of behavioral sensitization to morphine, this treatment inhibited the expression of morphine-induced conditioned locomotion (Experiment 2, Saline Challenge).

Behavioral sensitization is defined as an increase in locomotor activity caused by repeated drug administration. Behavioral sensitization has two distinct phases, induction and expression, marked, respectively, by neural changes that occur in VTA and long-term changes in neuronal function (Camarini and Pautassi, 2016). The behavioral sensitization paradigm has been used to investigate the abuse-related behavioral effects of different drug classes (Marinho et al., 2015; McGovern et al., 2015; Oliveira-Lima et al., 2015). The expression of behavioral sensitization induced by opioids seems to involve a hypersensitivity of neurons and dopaminergic receptors located in the mesoaccumbens region (Vanderschuren et al., 1999). The µ opioid receptors are highly expressed in the VTA, and neurocircuits that extend from the VTA to the NAcc are important in the rewarding effects of morphine (Kim et al., 2016).

Evidence indicates that cannabinoids and opioids act to activate mesolimbic dopaminergic transmission *via* a common pathaway, both *via* µ opioid receptors located in the VTA (Tanda et al., 1997; Maldonado et al., 2006). Therefore, treatment with morphine can modulate the dopaminergic system by mechanisms that involve both cholinergic and endocannabinoid circuits, particularly CB1 receptors located in the mesolimbic pathway NAcc (Khaleghzadeh-Ahangar and Haghparast, 2015). In agreement, studies have shown that CB1 receptor antagonists can block the development and expression of opioid-induced rapid-onset behavioral sensitization to morphine (Marinho et al., 2017) and morphine-induced conditioned place preference (Chaperon et al., 1998; Mas-Nieto et al., 2001; Singh et al., 2004; Azizi et al., 2009; Rashidy-Pour et al., 2013). The present study adds to this body of evidence by showing that treatment with the CB1 receptor antagonist rimonabant blocked the expression of morphine-induced behavioral sensitization.

The two doses of rimonabant used in the present study were selected based on previous studies from our laboratory showing that 10 mg/kg, but not 1 mg/kg, rimonabant blocked the expression of cocaine-induced behavioral sensitization using a similar experimental protocol (Marinho et al., 2017). We had also investigated the effects of rimonabant (0.3, 1, 3, and 10 mg/kg) on morphine-induced hyperlocomotion and single injection-induced sensitization to morphine (Marinho et al., 2015). Those findings showed similar effects for 0.3 and 1 mg/kg rimonabant, as well as 3 and 10 mg/kg rimonabant. Of note, in the study by Marinho et al. (2015), rimonabant was effective at blocking the acute and single injection-induced sensitization effects of morphine at 10 mg/kg, but not at 1 mg/kg. Therefore, in the present study we expected rimonabant to be effective at the highest dose only. However, important differences exist between the present and previous studies with morphine that could explain these discrepancies. In our previous study, rimonabant was administered before morphine, and 1 mg/kg was ineffective at blocking the development of acute morphine effects (Marinho et al., 2015). Also, Marinho et al. (2015) used a single injection of rimonabant, while in the present study animals were treated with rimonabant for eight consecutive days.

Studies suggest that the effects of CB1 receptors on drug abuse seem to be modulated by drug-environment conditioning. The context in which drugs are administered can interact with and mediate drug-related behavioral effects (Badiani and Robinson, 2004). In fact, this study shows that the effects of rimonabant on morphine-induced behavioral sensitization are dependent on the environmental context of rimonabant administration. Our findings are in agreement with studies showing that rimonabant only blocked cocaine-induced behavioral sensitization (Gerdeman et al., 2008) and ethanol-induced conditioned place preference (Silva et al., 2017) when it was administered in the same environmental context previously paired with the drug. The context-dependent effects of rimonabant have been attributed to the activity of the cannabinoid signaling as an occasion setter (Gerdeman et al., 2008; Silva et al., 2017). Based on this hypothesis (Anagnostaras and Robinson, 1996), after acquisition of morphine-induced behavioral sensitization, the open-field would predict drug administration and, thereby, be modulated by a CB1 receptor-mediated facilitating occasion setter. Therefore, inhibition of the endocannabinoid system by CB1 receptor antagonism would block the retrieval of the drug-associated contextual memory. This is corroborated by our findings showing that home-cage rimonabant treatment inhibited the expression of morphine-induced conditioned locomotion. The same effect was likely not observed after treatment with rimonabant in the drug-associated environment or in the novel environment because exposure to those environments led to extinction of morphine-induced conditioned locomotion. Therefore, in Experiments 1 and 3 both the control and the rimonabant groups failed to show conditioned locomotion to morphine during the Saline challenge.

Importantly, studies have shown that midbrain dopamine neurons act as a neurobiological substrate for encoding occasion setting properties (Aquili et al., 2020). Given the close interaction between dopamine and endocannabinoid signaling in the mesolimbic system, one could hypothesize that the effects of rimonabant in the present study may be mediated by occasion setting-induced activation of dopamine signaling when animals were exposed to the open-field apparatus during the treatment phase. The lack of rimonabant effect with home-cage treatment further corroborates this hypothesis. Of note, the fact that rimonabant also blocked the expression of morphine-induced behavioral sensitization when administered in a novel environment also suggest that dopamine activation is necessary for rimonabant to exert its effects. Studies have shown that acute and chronic responses to drugs of abuse can be modulated by environmental novelty (Caprioli et al., 2007), and exposure to novelty can potentiate the development of rapid-onset morphine-induced behavioral sensitization (Trombin et al., 2018). Importantly, these effects may be mediated by novelty-induced dopamine activation, as exposure to novelty can activate similar neuronal substrates that mediate the rewarding effects of drugs of abuse, particularly dopamine signaling within the mesolimbic system (Bardo et al., 1996). Therefore, in the present study exposure to the drug-associated environment (open-field) or to a novel environment (activity box), but not to a familiar environment (home-cage), would engender activation of dopamine signaling, allowing CB1 blockade to modulate drug-related behaviors.

While this dopaminergic theory might explain the lack of effects obtained with home-cage rimonabant treatment, the social context of home-cage treatments also may have contributed to our results. The social environment of group-housed adult mice often requires animals to adopt social defense and subordinate behaviors (Miczek et al., 2009), which can be a distress factor and promote adaptive behaviors and changes in corticosterone levels (McQuaid et al., 2013). Even brief episodes of defense-related distress can trigger behavioral sensitization or prolong self-administration of opioids, and daily episodes of social defeat also result in cross-sensitization to morphine-induced hyperactivity (Miczek et al., 2009). Together with studies showing that CB1 gene disruption (knock-out) promotes aggressive home-cage behavior in mice (Haller et al., 2004), these findings suggest that the social dynamics of the treatment environment of Experiment 2 (home-cage) also may have prevented the therapeutic effects of rimonabant from emerging.

In summary, treatment with the CB1 receptor antagonist rimonabant blocked the expression of behavioral sensitization induced by morphine in a context-dependent manner. Rimonabant was effective when administered in the drug-associated environment or in a novel environment, but not when given in the home-cage. The context-dependent effects of rimonabant may be related not only to the occasion-setting properties exercised by endocannabinoid signaling, but also to the distress caused by the social interaction of mice in the housing environment. Of note, the present study presents some limitations, including the lack of sex differences investigation, and the use of a single opioid drug and cannabinoid receptor antagonist, limiting our ability to generalize these findings to other drugs and conditions. Despite these limitations, our findings are in agreement with the growing literature suggesting an interaction between endocannabinoid and opioid systems, emphasize that the endocannabinoid system remains a promising target for OUD treatment, but the treatment environment must be taken into consideration when developing treatment strategies.

## Data Availability

The raw data supporting the conclusion of this article will be made available by the authors, without undue reservation.
